# MicroRNA-449a enhances radiosensitivity by downregulation of c-Myc in prostate cancer cells

**DOI:** 10.1038/srep27346

**Published:** 2016-06-02

**Authors:** Aihong Mao, Qiuyue Zhao, Xin Zhou, Chao Sun, Jing Si, Rong Zhou, Lu Gan, Hong Zhang

**Affiliations:** 1 Institute of Modern Physics, Chinese Academy of Sciences, Lanzhou 730000, PR China; 2 School of Nuclear Science and Technology, Lanzhou University, Lanzhou 730000, PR China; 3 Institute of Gansu Medical Science Research, Lanzhou 730050, PR China; 4 University of Chinese Academy of Sciences, Beijing 100039, PR China; 5 Key Laboratory of Heavy Ion Radiation Medicine of Chinese Academy of Sciences, Lanzhou 730000, PR China; 6 Key Laboratory of Heavy Ion Radiation Medicine of Gansu Province, Lanzhou 730000, PR China

## Abstract

MicroRNAs (miRNAs) have been reported to be involved in DNA damage response induced by ionizing radiation (IR). c-Myc is reduced when cells treated with IR or other DNA damaging agents. It is unknown whether miRNAs participate in c-Myc downregulation in response to IR. In the present study, we found that miR-449a enhanced radiosensitivity *in vitro* and *in vivo* by targeting c-Myc in prostate cancer (LNCaP) cells. MiR-449a was upregulated and c-Myc was downregulated in response to IR in LNCaP cells. Overexpression of miR-449a or knockdown of c-Myc promoted the sensitivity of LNCaP cells to IR. By establishing c-Myc as a direct target of miR-449a, we revealed that miR-449a enhanced radiosensitivity by repressing c-Myc expression in LNCaP cells. Furthermore, we showed that miR-449a enhanced radiation-induced G2/M phase arrest by directly downregulating c-Myc, which controlled the Cdc2/CyclinB1 cell cycle signal by modulating Cdc25A. These results highlight an unrecognized mechanism of miR-449a-mediated c-Myc regulation in response to IR and may provide alternative therapeutic strategies for the treatment of prostate cancer.

c-Myc is one of the first oncogenes to be identified and its overexpression at the RNA and protein levels has subsequently been linked to a wide variety of human cancers^1^. Overexpression of the c-Myc protein or c-Myc gene has been shown in 80% of breast cancers, 70% of colon cancers, 90% of gynecological cancers, 50% of hepatocellular carcinomas and a variety of hematological tumors. It is estimated that approximately 100 000 US cancer deaths per year are associated with changes in the c-Myc gene or its expression^2^. In prostate cancer, c-Myc is involved in disease progression and the presence of its amplification is strongly associated with high histological grade and worse prognosis^3^,^4^,^5^,^6^. Recent evidence shows that approximately 30% of prostate cancer specimen exhibits c-Myc amplification^7^,^8^. In addition, overexpression of c-Myc mRNA in primary prostate cancer predicates biochemical recurrence^9^ and that increased copy number for c-Myc strongly predicts systemic progression and patient death^10^. Furthermore, c-Myc amplification not only contributes to the genesis and progression of most human tumors, but affects the outcome of cancer radio- or chemotherapy^11^,^12^. Indeed, a series of reports have demonstrated that the overexpression of c-Myc contributed to cancer radioresistance^13^,^14^,^15^,^16^,^17^. Thus, targeting c-Myc could be a potential strategy against prostate cancer.

MicroRNAs (miRNAs) are evolutionarily conserved, endogenous, small noncoding RNAs that regulate the stability and translation of target mRNA by primarily binding to the 3′-UTR^18^. In the last decade, an abundance of *in vivo* and *in vitro* studies have demonstrated that miRNAs play a critical role in carcinogenesis and cancer progression^19^,^20^,^21^ and deregulation of miRNAs has been observed in various human cancers^22^. Thus, some miRNAs have been proposed as novel potential targets for cancer therapy^23^,^24^. Futhermore, recent evidence has confirmed that there is significant crosstalk between c-Myc and miRNA. Several miRNAs have been identified as regulators of c-Myc^25^,^26^,^27^,^28^,^29^. Interestingly, it was found that miR-34a suppressed the malignancy of human prostate cancer cells by modulating the c-Myc transcriptional complex^30^. During oncogene-induced senescence, miR-34a was also found to target c-Myc^31^. In addition, the miR-34b/c cluster can directly target the c-Myc transcript in prostate cancer cells^32^.

MicroRNA-449a (miR-449a) is the best characterized member of the microRNA-449 family (miR-449b, and miR-449c), which contains the same seed sequences as the miRNA-34 family (miR-34a, miR-34b, miR-34c)^33^. Due to high similarity in the seed sequence, these six miRNAs form a functionally related miRNA family. MiR-449a is deregulated in various types of cancers, including prostate cancer^34^,^35^,^36^. Overexpression of miR-449a can induce significant cell senescence and inhibit cancer cell growth, migration and invasion by directly targeting oncogenes^34^,^37^,^38^,^39^,^40^. Although miR-34c has been shown to negatively regulate c-Myc in response to DNA damage^41^, whether miR-449a and the other five members have distinct or overlapping targets is yet to be elucidated and the precise role of miR-449a in the response to IR is unknown. Furthermore, functionally, miR-449a is a key miRNA that inhibits cancer cell proliferation, invasion and migration by targeting components that promote cell proliferation or have oncogenic potential. To date, several targets of miR-449a have been identified, including MET, GMNN, CCNE2, SIRT1^42^, HDAC1^34^, CKD6, CDC25A^37^ and E2F^43^. The results of these studies suggested that miR-449a may have potential application in tumor treatment.

In this study, we showed that miR-449a enhanced the radiosensitivity of prostate cancer *in vitro* and *in vivo* by targeting c-Myc in prostate cancer (LNCaP) cells. MiR-449a was upregulated and c-Myc was downregulated in response to IR in LNCaP cells. Either overexpression of miR-449a or knockdown of c-Myc enhanced radiation-induced G2/M phase arrest and sensitized LNCaP cells to IR. By establishing c-Myc as a direct target of miR-449a, we revealed that miR-449a enhanced radiosensitivity by repressing c-Myc expression in LNCaP cells. Moreover, we found that miR-449a-targeted c-Myc controlled the Cdc2/CyclinB1 cell cycle signal by modulating Cdc25A and regulated the cell cycle progress in prostate cancer cells following IR. This study revealed a previously unrecognized function of miR-449a-mediated c-Myc regulation in response to IR, Which highlighted an unrecognized mechanism of miR-449a-mediated c-Myc regulation in response to IR and may provide alternative therapeutic strategies for the radiation treatment of prostate cancer.

## Results

### MiR-449a is upregulated in response to IR in LNCaP cells

**Figure 1:**
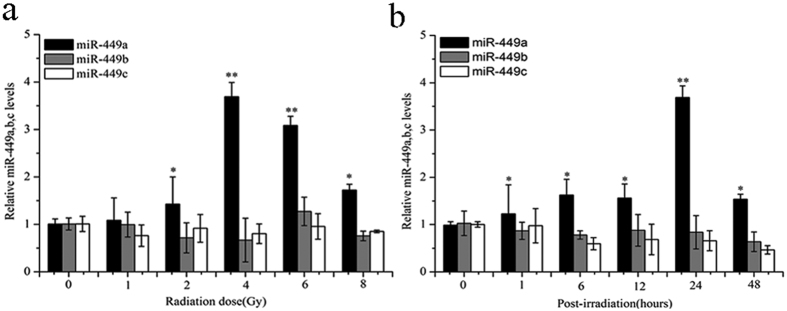
MiR-449a expression is upregulated in LNCaP cells in response to IR. (**a**) Relative expression levels of miR-449a, b and c in LNCaP cells after exposure to the indicated doses of X-rays 24 h. (**b**) Relative expression levels of miR-449a, b and c in LNCaP cells at the indicated time points after exposure to 4Gy of X-rays. Data are representative of at least three independent experiments. *p < 0.05 and **p < 0.01 versus control group.

To assess alterations in expression of the functionally related miRNA family in response to IR, miR-449 family microRNAs were determined using quantitative RT-PCR analysis. As shown in Fig. 1a,b, of the three microRNAs, miR-449a was markedly upregulated in LNCaP cells following IR and its expression reached the maximum level 24 h post-IR of 4 Gy X-rays. These results demonstrated that IR treatment significantly altered miR-449a expression in LNCaP cells. MiR-449a may play a role in determining the outcome of radiation treatment.

### Knockdown of miR-449a suppresses radiation-induced growth arrest

**Figure 2:**
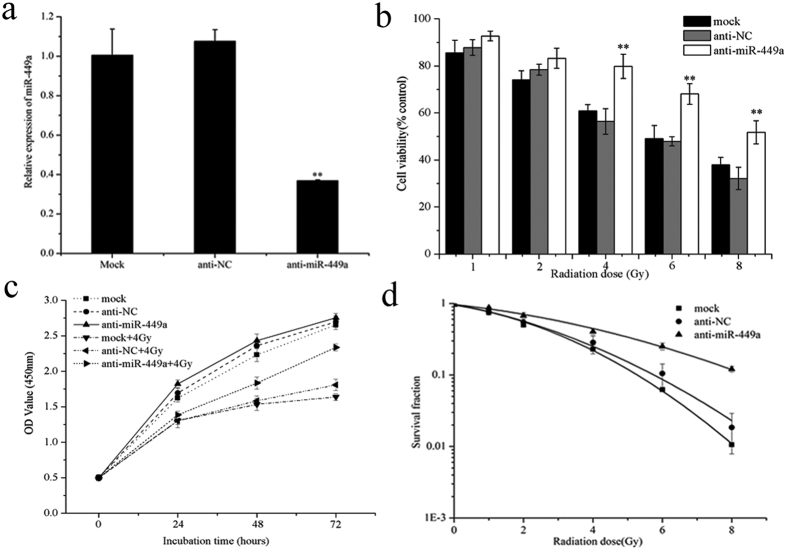
Knockdown of microR-449a reduces radiosensitivity of LNCaP cells. (**a**) MiR-449a expression after transfection with anti-miR-449a or negative control (NC) into LNCaP cells. (**b**) Cell viability, (**c**) cell proliferation and (**d**) colony formation of LNCaP cells transfected with anti-miR-449a or NC after IR. Data are representative of at least three independent experiments. *p < 0.05 and **p < 0.01 versus the control group.

To study the radiobiological effects of miR-449a in prostate cancer cells, we first suppressed miR-449a expression by transiently transfecting LNCaP cells with miR-449a antagomir (anti-miR-449a) and a non-targeting sequence (negative control, NC). Transient transfection of anti-miR-449a markedly inhibited miR-449a expression levels in LNCaP cells (Fig. 2a). Knockdown of miR-449a increased cell viability (Fig. 2b) and attenuated the growth inhibitory effects induced by IR (Fig. 2c). In addition, a colony formation assay showed that reduced miR-449a expression increased the survival fraction of LNCaP cells after IR treatment (Fig. 2d). Thus, decreased miR-449a expression attenuated cellular response to IR and reduced the sensitivity of LNCaP cells to IR.

### Overexpression of miR-449a enhances radiosensitivity of LNCaP cells

**Figure 3:**
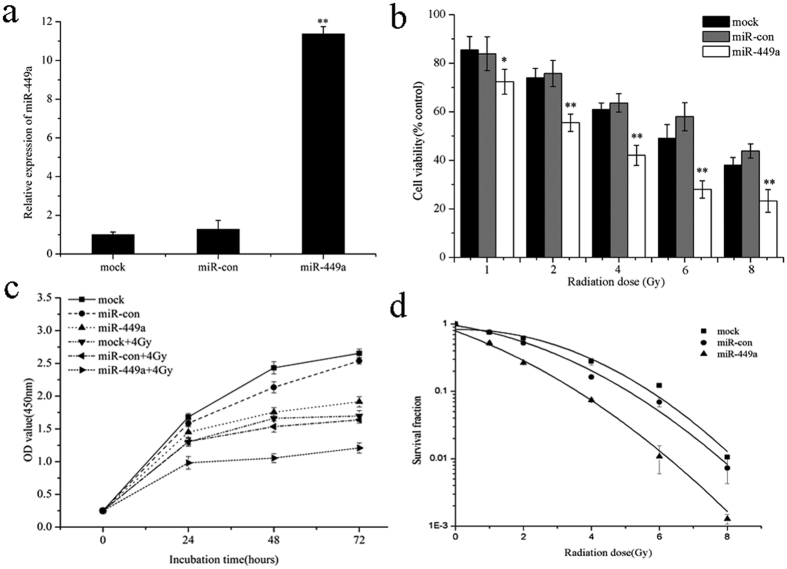
Overexpression of miR-449a enhances radiosensitivity of LNCaP cells. (**a**) The changes IN miR-449a expression after transfection with the miR-449a or negative control expression plasmid. (**b**) Cell viability, (**c**) cell proliferation and (**d**) colony formation of miR-449a overexpressed cells treated with X-rays irradiation. Data are representative of at least three independent experiments. *p < 0.05 and **p < 0.01 versus the control group.

To further examine the role of miR-449a in response to IR, a miRNA expression plasmid containing a primary miR-449a sequence was used to overexpress miR-449a in LNCaP cells. Transfection of miR-449a expression plasmid significantly increased miR-449a levels (Fig. 3a). Overexpression of miR-449a combined with X-ray irradiation had greater effect on cell viability and growth than radiation alone (Fig. 3b,c). Results of the colony formation assay also showed that the survival fraction of LNCaP cells transfected with miR-449a was significantly lower than that of control cells after IR (Fig. 3d). These data demonstrated that elevated miR-449a decreased cell viability and suppressed cell proliferation after IR, resulting in increased sensitivity of LNCaP cells to IR.

### MiR-449a suppresses c-Myc expression by targeting the 3′-UTR of c-Myc mRNA

**Figure 4:**
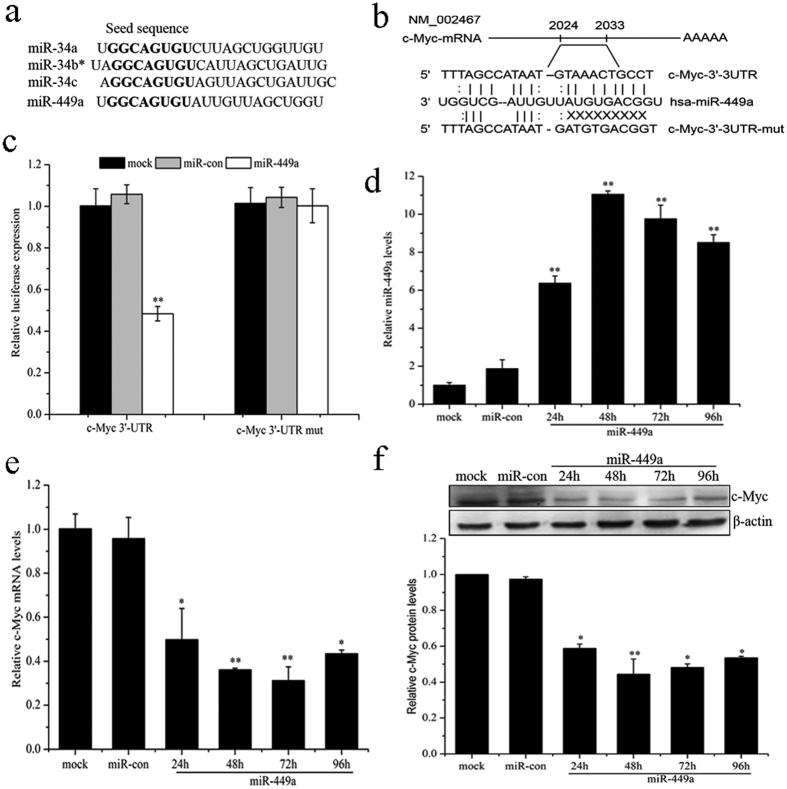
MiR-449a suppresses c-Myc expression by targeting the 3′-UTR of c-Myc mRNA. (**a**) Comparison of human miR-449a and miR-34a, b and c sequences. Seed sequences are shown in bold. (**b**) Putative miR-449a binding site within the human c-Myc 3′-UTR is shown at the top. The sequences of mature miR-449a aligned to the target site and the 3′-UTR mutated in the miR-449a seed-pairing sequence was shown below. (**c**) Luciferase reporter assay was performed 48 h after co-transfection in LNCaP cells with Wt c-Myc or Mut c-Myc vectors together with miR-449a or negative control. (**d**) qRT-PCR analysis of miR-449a expression at the indicated time points after transfection with miR-449a into LNCaP cells. (**e**) c-Myc expression is repressed by miR-449a at the mRNA level. qRT-PCR was conducted to quantify c-Myc expression at the indicated time point after transfection with miR-449a into LNCaP cells. (**f**) c-Myc expression is repressed by miR-449a at the protein level. Western blotting was performed at the indicated time points after transfection with miR-449a into LNCaP cells. Data are representative of at least three independent experiments. *p < 0.05 and **p < 0.01 versus the control group.

The cellular functions of miRNAs are revealed through their target genes. Oncogene c-Myc has identified binding sites for miR-34a and miR-34b/c in their 3′-UTR^30^,^32^,^41^. Interestingly, human miR-449a and miR-34a, miR-34b/c share identical seed sequences (Fig. 4a). Target Scan predictions also revealed that a conserved sequence in the 3′-UTR of c-Myc mRNA has a perfect match to the seed region of miR-449a (Fig. 4b). To identify c-Myc mRNA is a direct target of miR-449a, we transfected miR-449a overexpressed LNCaP cells together with pGL3-c-Myc 3′-UTR or pGL3-c-Myc 3′-UTR mutant. As shown in Fig. 4c, overexpression of miR-449a significantly inhibited the luciferase activity of the wild-type c-Myc 3′-UTR reporter, but not that of the mutant reporter, which suggested miR-449a targeted c-Myc mRNA through its 3′-UTR. To determine whether c-Myc levels are affected by miR-449a expression, we further assayed c-Myc mRNA and protein levels. qRT-PCR revealed that miR-449a significantly increased, whereas c-Myc mRNA markedly decreased in cells transfected with miR-449a (Fig. 4d,e). The c-Myc protein level was markedly suppressed in cells transfected with miR-449a (Fig. 4f), which was consistent with the qRT-PCR results. The targeted inhibition of c-Myc by miR-449a was further confirmed by downregulation of c-Myc at the both mRNA and protein level. Moreover, we also found that miR-449a downregulated the expression of c-Myc in PC-3 and DU-145 cell lines (Supplementary Fig. S1a,b). These results indicated that miR-449a directly regulated the expression of c-Myc by targeting 3′-UTR of its mRNA.

### Response of miR-449a and c-Myc to IR in LNCaP cells

It has been reported that c-Myc is upregulated^44^ and miR-449a is downregulated in prostate cancer cells^34^. Either downregulation of c-Myc or overexpression of miR-449a suppressed the proliferation, and resulted cell cycle arrest and senescence in prostate cancer cells. According qRT-PCR results of prostate cancer tissue samples and matched normal prostate tissue samples (5 pairs of prostate tissue samples), we found that miR-449a/b/c was significantly down-regulated in prostate cancer tissue compared with normal prostate tissues (*P* = 0.000389, *P* = 0.00108 and *P* = 0.000128, respectively), while c-Myc was upregulated in prostate cancer tissues(*P* = 0.000254; Supplementary Fig. S2). However, whether they participate in modulating radiobiological effects in the prostate cancer cells are still unknown.

**Figure 5:**
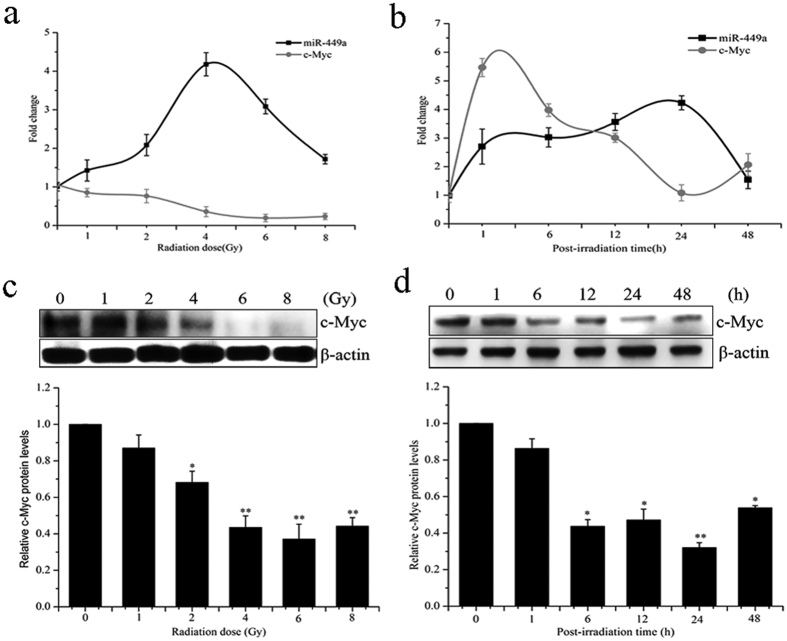
Response of miR-449a and c-Myc to IR in LNCaP cells. (**a**) Expression levels of miR-449a and c-Myc mRNA at the indicated doses of X-rays irradiation 24 h. (**b**) Expression levels of miR-449a and c-Myc mRNA at the indicated time points after irradiation with 4 Gy of X-rays. (**c**) c-Myc protein level at the indicated doses of X-rays irradiation 24 h. (**d**) c-Myc protein level at the indicated time points after irradiation with 4 Gy of X-rays. Data are representative of at least three independent experiments. *p < 0.05 and **p < 0.01 versus the control group.

To investigate the correlation between miR-449a and c-Myc in response to IR in LNCaP cells, miR-449a expression and c-Myc mRNA levels were analyzed by qRT-PCR after IR. As shown in Fig. 5a,b, the expression of miR-449a gradually increased after exposure to 1- 4 Gy of X-ray, and then decreased, while c-Myc mRNA gradually reduced by treatment of LNCaP cells with IR. A negative correlation between miR-449a and c-Myc was observed. The c-Myc protein levels were examined by western blot and the c-Myc protein levels were markedly reduced in LNCaP cells at 24 h following IR (Fig. 5c,d). These results suggested that miR-449a and c-Myc were negatively correlated in response to IR in LNCaP cells.

### Knockdown of c-Myc sensitizes LNCaP cells to IR

**Figure 6:**
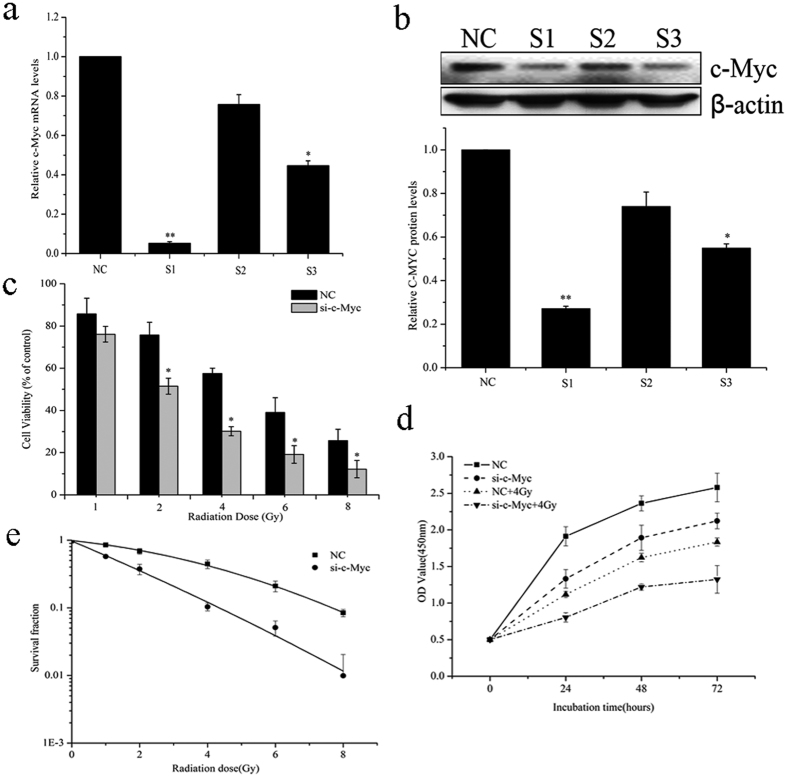
Knockdown of c-Myc sensitizes LNCaP cells to IR. (**a**) c-Myc mRNA expression levels and (**b**) c-Myc protein level after transfection with siRNA-c-Myc or negative control (NC) into LNCaP cells. (**c**) Cell viability, (**d**) cell proliferation and (**e**) colony formation of c-Myc-knockdown (si-c-Myc) cells treated with X-rays irradiation. Data are representative of at least three independent experiments. *p < 0.05 and **p < 0.01 versus the control group.

As c-Myc has been shown to be a target of miR-449a and overexpression of miR-449a enhanced the sensitivity of LNCaP cells to IR, we subsequently attempted to determine whether miR-449a sensitizes LNCaP cells to IR by directly targeting c-Myc. To validate this, c-Myc was knocked down by a specific siRNA in LNCaP cells and cell viability, cell proliferation and colony formation ability were evaluated after IR. As shown in Fig. 6a,b, both mRNA expression and protein level of c-Myc were significantly suppressed by c-Myc siRNA1 (S1), compared to the negative control (NC). Thus siRNA1 was selected to knockdown c-Myc expression in LNCaP cells and is referred to as si-c-Myc. After IR, LNCaP cells transfected with si-c-Myc had a significant lower viability than the NC group (Fig. 6c). Cell proliferation was strongly inhibited by 4Gy of X-ray irradiation in LNCaP cells transfected with si-c-Myc (Fig. 6d). The survival fractions of LNCaP cells transfected with si-c-Myc were markedly lower than those of NC cells (Fig. 6e). In addition, knockdown of c-Myc also enhanced radiosensitivity in other prostate cancer cell lines, PC-3 and DU-145(Supplementary Fig. S3). These data suggested that knockdown of c-Myc increased the sensitivity of prostate cancer cells to IR.

Taken together, the above results confirmed that miR-449a enhanced the radiosensitivity of prostate cancer cells by targeting regulation of c-Myc.

### MiR-449a directly targeting c-Myc enhances radiation-induced G2/M arrest

**Figure 7:**
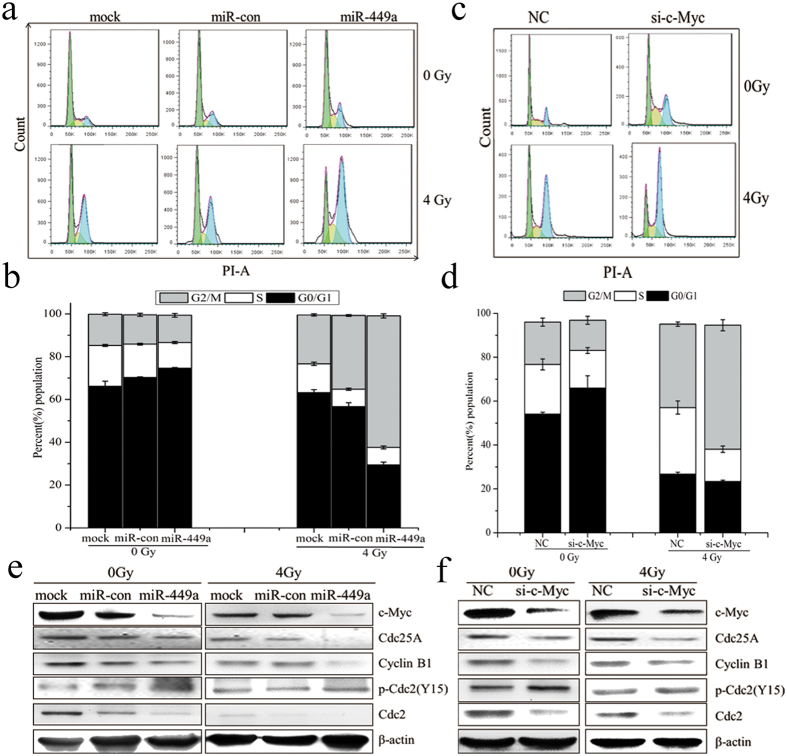
MiR-449a enhances radiation-induced G2/M phase arrest by repressing c-Myc in LNCaP cells. (**a**) Cell cycle distribution in LNCaP cells transfected with miR-449a or negative control (NC) expression plasmid and treated with 4Gy of X-rays at 24 h. (**b**) The percentage of LNCaP cells in each phase at 24 h after exposure to 4Gy of X-rays. (**c**) Cell cycle distribution in LNCaP cells transfected with siRNA-c-Myc or negative control (NC) after exposure to 4Gy of X-rays at 24 h. (**d**) The percentage of LNCaP cells in each phase after exposure to 4Gy of X-rays 24 h. (**e**) Overexpression of miR-449a reduces Cdc25A, Cdc2/Cyclin B1 expression and increase Cdc2 phosphorylation in response to IR. (**f**) Knockdown of c-Myc reduces Cdc25A, Cdc2/Cyclin B1 expression and increases Cdc2 phosphorylation in response to IR. Data are representative of at least three independent experiments.

It has been reported that the alteration of cell cycle progression post-irradiation closely correlated with sensitivity^45^. To further investigate the mechanism of miR-449a in response to IR, cell cycle of LNCaP cells transfected miR-449a or si-c-Myc was analyzed by flow cytometry 24 h after IR. As shown in Fig. 7a,b, the G2/M phase arrest was more pronounced in miR-449a overexpressed LNCaP cells than in control cells. As c-Myc is a direct target of miR-449a and plays an essential role in the regulation of cell cycle progression, we evaluated the consequence of si-c-Myc in LNCaP cells. As shown in Fig. 7c,d, knockdown of c-Myc similarly enhanced radiation-induced G2/M phase arrest. These results suggested that overexpression of miR-449a enhanced radiation-induced G2/M arrest by downregulation of c-Myc in LNCaP cells.

To investigate the possible molecular mechanism of miR-449a in suppressing cell proliferation and enhancing G2/M phase arrest induced by IR, several cell cycle- related proteins were examined by western blot. As shown in Fig. 7e, ectopic miR-449a markedly decreased the levels of c-Myc, Cdc25A, Cyclin B1 and Cdc2 in cell cycle transition, and significantly increased the phosphorylation of Cdc2. In response to IR, c-Myc, Cdc25A, Cyclin B1 and Cdc2 further decreased in miR-449a overexpressed cells. Knockdown of c-Myc by specific siRNA resulted in similar results (Fig. 7f). These results suggested that, after IR, miR-449a suppressed G2/M cycle signal resulting in the accumulation of cells in G2/M phase by repressing c-Myc expression. Furthermore, Suppression or degradation of Cdc25A in response to IR contributed to cells arrest at G2/M, which then repressed the genes needed for mitosis^46^. As Cdc25A was known to affect phosphorylation of pRb and E2F1 expression^34^,^37^, we therefore analyzed the levels of phosphorylated pRb and E2F1. Introduction of miR-449a resulted in dramatically inhibition of pRb phosphorylation and reduced E2F1 expression (Supplementary Fig. S4).These data suggested that miR-449a enhanced radiation-induced G2/M phase arrest by regulating c-Myc/Cdc25A pathway to control the Cyclin B1/Cdc2 activity.

### MiR-449a enhances radiosensitivity of prostate cancer in xenograft models

**Figure 8:**
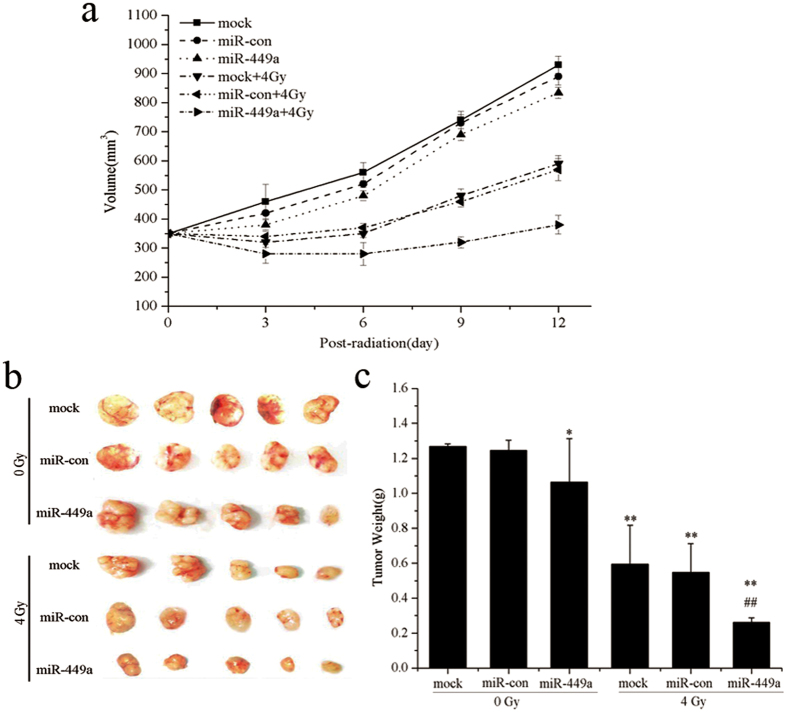
MiR-449a enhances radiosensitivity of prostate cancer in xenograft models. (**a**) Tumor sizes of nude mice carrying LNCaP xenografts treated with 4 Gy X-ray radiation as indicated. Error bar represent SD (n = 5). (**b**) and (**c**) Tumors were excised and weighed at the end of the experiment (26 days). *p < 0.05 and **p < 0.01 versus the control group, ^##^p < 0.01 versus the control group treated with 4Gy X-ray radiation.

To further identify that miR-449a enhances radiosensitivity of prostate cancer *in vivo*, we performed xenograft studies. We used human prostate cancer LNCaP cells and GV214-miR-con or GV214-miR-449a-transfected LNCaP cells to verify the radiosensitivity of tumor in nude mouse followed 4Gy X-ray radiation. As shown in Fig. 8a,b, tumor growth was not controlled in control group (mock and miR-con). After treatment with 4Gy X-ray radiation, continuous growth of tumors was still observed. However, the tumor growth rates in mice bearing GV214-miR-449a-transfected LNCaP tumor(miR-449a) were evidently slower than that of control group (Fig. 8a), and tumor weights were significantly lighter in miR-449a treated animals compared to the control group (Fig. 8b,c). These data suggested that these miR-449a significantly suppressed tumor growth and reduced the tumor weights in tumor-bearing mice after radiation treatment.These results were consistent with the radiosensitization effects of miR-449a.

## Discussion

c-Myc is a DNA-binding protein that belongs to the MYC/MAD/MAX family of basic-helix-loop-helix-zipper (bHLHz) proteins^47^. As a transcription factor, the c-Myc protein regulates a variety of cellular processes including cell growth and proliferation, cell-cycle progression, transcription, differentiation, apoptosis and cellular motility^48^ and knockdown of c-Myc by siRNA or some of its effectors, miRNAs, can significantly reduce cancer cell growth and increase the apoptosis *in vitro* and suppresses tumorigenicity *in vivo*^44^. MiR-449a is a well-known tumor suppressor miRNA which islocated on chromosome 5 (5q11.2) in the second intron of the Cdc20b gene^49^. Studies showed that endogenous miR-449a expression is significantly lower in many cancers, compared with the matched normal tissue samples. It has been demonstrated that overexpression of miR-449a can induce significant cell senescence and inhibit cancer cell growth, migration and invasion by directly targeting oncogenes^34^,^37^,^38^,^39^,^40^. In this study, we identified that c-Myc is a target of miR-449a and miR-449a suppressed c-Myc expression by directly binding the 3′-UTR of c-Myc mRNA. These results indicated that miR-449a not only functions as a tumor suppressor by directly targeting some oncogenes mRNA, but also by regulating the transcription factor c-Myc expression to indirectly affect the expression of other tumor suppressor genes.

Cdc25A, itself a proto-oncogene, is a physiologically relevant transcriptional target of c-Myc^50^. Studies pointed to a potential role for Cdc25A as a mediatior of Myc function. Myc stimulates the activity of cyclin-E and cyclin-D1-dependent kinases (Cdk2 and Cdk4), without altering the levels of Cdk2, Cdk4, cyclin-E or cyclin D1. Cdc25A is a well-established activator of cyclin-dependent kinases (CDKs) by removing inhibitory phosphates from the tyrosine and threonine residues, resulting in CDKs activation and cell-cycle progression. A key substrate of both Cdk4 and Cdk2 kinases is the retinoblastoma protein (Rb), and induction of Myc leads to Rb phosphorylation and inactivation^51^, presumably by the activated CDKs. In the present study, we showed that miR-449a markedly decreased the levels of c-Myc, Cdc25A, resulting in dramatically inhibition of pRb phosphorylation and reduced E2F1 expression. It has also been reported that miR-449a can directly target and inhibit oncogenic CDK6 and Cdc25A, resulting in pRb dephosphorylation^37^. Upon Rb phosphorylation, E2F proteins are released to activate downstream gene expression. Therefore, these reports, in combination with our data, defined miR-449a enhanced radiation-induced growth inhibition and G2/M arrest by directly or indirectly targeting Cdc25A to control the Rb/E2F1 activity.

**Figure 9:**
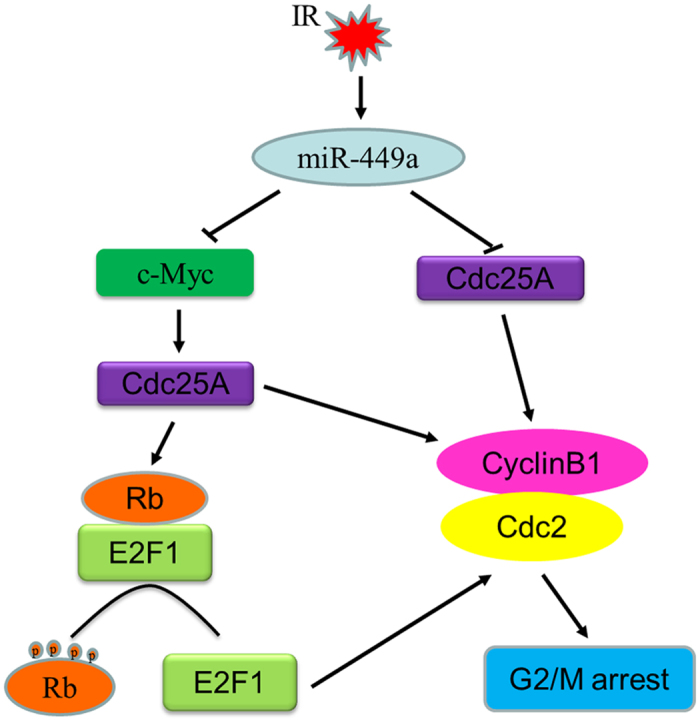
A schematic diagram illustrating miR-185 and ATR function in radiation response. MiR-449a is induced in LNCaP cells following exposure to ionizing radiation. Elevated miR-449a represses c-Myc expression by by directly targeting the 3′-UTR of c-Myc mRNA. Cdc25A, as a mediatior of Myc function, is suppressed or degraded in response to DNA damage induced by ionizing radiation, resulting in dramatical inhibition of pRb phosphorylation and reduced E2F1 expression. Upon Rb phosphorylation, E2F proteins are released to activate downstream gene Cdc2. Cdc25A itself is a direct target of miR-449a and is capable of binding to cyclinB1 and activate Cdc2/cyclinB1 complexes. Consequently, miR-449a enhanced radiation-induced G2/M phase arrest and sensitizes cancer cells to ionizing radiation by downregulating c-Myc/Cdc25A pathway or directly repressing Cdc25A to modulate the Cdc2/cyclinB1 complexes.

Moreover, Cdc25A not only regulates the G1- to S-phase transition, but also have a role in regulating the G2- to M-phase transition. Cdc25A is capable of binding to cyclin B1 and can activate Cdc2/cyclin B1 complexes *in vitro*, and overexpression of Cdc25A accelerates entry into mitosis, and expression of a phosphatase-inactive mutant of Cdc25A delays entry into mitosis^46^. In this study, we found that overexpression of miR-449a or knockdown of c-Myc caused downregulation of Cdc25A, and markedly decreased CyclinB1 and Cdc2 activation and increased phosphorylation of Cdc2. These results suggested that miR-449a enhanced radiation-induced G2/M phase arrest by regulating c-Myc/Cdc25A pathway to modulate the Cdc2/cyclinB1 complexes. Based on our data and above literatures, a simple model can be created linking miR-449a to c-Myc expression and sensitizing prostate cancer cells to X-ray radiation (Fig. 9).

Prostate cancer is the second most common cancer worldwide in men, with an estimated 1,100,000 cases and 307,000 deaths in 2012 (World Health Organization). In the United States, there will be an estimated 220,800 cases and 27,540 deaths in 2015^52^. Currently, radiotherapy is one of the most common definitive treatment options for localized prostate cancer. However, it is by no means innocuous and local recurrences and radioresistance remain significant clinical problems^53^. Therefore, how to reduce tumor radioresistance and improve tumor radiosensitivity is a hot topic in the radiotherapeutic field of prostate cancer. In this study, we found miR-449a was upregulated and c-Myc was downregulated in response to IR in LNCaP cells. By establishing c-Myc as a direct target of miR-449a, we revealed that miR-449a directly regulated the expression of c-Myc by binding the 3′-UTR of its mRNA. Furthermore, Overexpression of miR-449a or knockdown of c-Myc sensitized prostate cancer cells to X-ray radiation along with a significantly growth inhibition and cell cycle arrest. In this study, besides demonstrating that there is a a negative correlation between miR-449a and c-Myc, we also found that these two regulating factors responded to X-ray radiation. Thus, identifying the connection between miR-449a and c-Myc in response to IR contributes to a better understanding of prostate carcinogenesis and prostate cancer radiotherapy.

Furthermore, androgen receptor (AR) is critical for the development and progression of prostate cancer (PCa) and AR inhibition represents the first-line therapeutic modality for patients with advanced disease^54^. Endocrine therapies directed toward reducing serum androgens and inhibition of AR initially block PCa growth, but often fatal castration-resistant disease develops^55^. Therefore, it is critically important to understand the regulation of its expression and to find novel ways of inhibiting this pathway. Recently, studies demonstrated that some miRNAs might play a role in AR-mediated signals in prostate cancer progression. For example, Shi *et al.* found AR could function through up-regulating the miR-125b expression to suppress Bak1 expression to promote prostate cancer progression^56^. Ribas *et al.* also found that AR could directly bind to miR-21 promoter to exert its influence on the prostate cancer growth^57^, and Murata *et al.* reported that miR-148a was an androgen-responsive miRNA that could promote prostate LNCaP cell growth via repressing its target CAND1 expression^58^. Interestingly, Ostling *et al.* showed that the miR-449a exert regulation on AR primarily through the 3′-UTR of AR mRNA to decrease the levels of AR^59^. While c-Myc expression is up-regulated rapidly by AR signaling in prostate cancer cells^60^. Direct AR regulation of MYC cooperates with AR-mediated activation of HER2/HER3 signaling. Elevated c-Myc expression, in turn, reinforces the transcriptional activation of androgen-responsive genes^61^. Thus, we speculate that overexpression of miR-449a or knockdown of c-Myc may inactivate AR signaling and then increase the radiosensitivity of prostate cancer cell to ionizing radiation.

In summary, our study reported here demonstrated that miR-449a enhanced the sensitivity of LNCaP cells to IR by directly targeting c-Myc, which controlled the Cdc2/cyclinB1 cell cycle signal by modulating Cdc25A/Rb/E2F pathway. These results enriched the complex relationship of miRNA and tumorgenesis. Furthermore, we found that both miR-449a and c-Myc responded to irradiation and either overexpression of miR-449a or knockdown of c-Myc sensitized LNCaP cells to irradiation. These findings highlighted an unrecognized mechanism of miR-449a-mediated c-Myc regulation in response to IR, which provides a support for the combination of ionizing radiation with miRNAs regulation as a therapeutic strategy for patients with prostate cancer.

## Methods

### Cell culture and irradiation treatment

The human prostate cancer lines, LNCaP, PC-3 and DU-145 cells were purchased from the Cell Bank of the Chinese Academy of Sciences (Shanghai, China). Cells were maintained in RPMI-1640 medium (GIBCO, USA) supplemented with 10% fetal bovine serum (FBS) (Hyclone, USA). Cells were cultured in a humidified atmosphere with 5% CO_2_ at 37 °C.

X-rays were generated by an X-ray machine (Faxitron RX-650, USA) with 100 kVp. The dose rates for delivering was 0.835 Gy/min. Cells in exponential growth were irradiated at room temperature, and non-irradiated culture cells were handled in parallel with the irradiated samples.

### Tissues

Tumor and corresponding normal prostate tissue specimens were obtained from patients who underwent curative resection at the Gansu Provincial Cancer Hospital, with written informed consent obtained from all subjects and approval by the Bioethics Committee of Gansu Provincial Cancer Hospital. And the methods were carried out in accordance with the approved guidelines. None of the patients had received chemotherapy or radiotherapy prior to surgery. All of the tumor and macroscopically normal prostate tissue samples were obtained at the time of surgery, and were rapidly frozen in liquid nitrogen and stored at −80 °C until analysis.

### RNA isolation, reverse transcription, and qRT-PCR

Total RNA was extracted from prostate tissue or LNCaP cells using Trizol (Takara, Japan). For miRNAs qRT-PCR, a total of 10 ng of RNA was transcribed into cDNA using the Taqman miRNA reverse transcription kit (Takara, Japan). And real-time PCR was performed with specific sense primers (Supplementary Table S1) and general antisense primer supplied by miRNA PrimeScript RT Enzyme Mix Kit (Takara, Japan) according to the manufacturer’s instructions. Quantitative c-myc gene-expression analysis was performed with specific primers (Supplementary Table S2) by using one step SYBR PrimeScript plus RT-PCR kit (Takara, Japan) according to the manufacturer’s instructions. MiRNAs and mRNA expression were normalized to RNU6 and GAPDH, respectively, using the 2^−∆∆Ct^ method.

### Cell transfection

Overexpressing miR-499a and miR-con (Supplementary Table S3) was generated by plasmid transduction using GV214 (GeneChem Co., Ltd, Shanghai, China). MiR-449a antagomir (anti-miR-449a) and non-targeting sequence (negative control, anti-NC) (Supplementary Table S4) were synthesized (RiboBio, Guangzhou, China). SiRNA that target c-Myc and its negative control (NC) (Supplementary Tables S4) were purchased from Invitrogen (USA). For transfection, the cells were plated on an antibiotic free growth medium at 30–40% confluence approximately 24 h before transfection. Transfection was performed with Trans IT^®^-2020 Transfection Reagent (Mirus Bio LLC, USA) according to manufacturer’s protocol. The medium was replaced with new culture medium 6 h after transfection.

### Cell viability assay

The thiazolyl blue tetrazolium blue (MTT)-based cell viability assay was carried out as described. 1 × 10^3^ cells were seeded into a 96-well plate with 6 repeat for each condition. After 12 h, the cells were treated with 4 Gy X-rays. Approximately 48 h after IR, 10 μl MTT (5 mg/ml) (Sigma, USA) was added to each well and incubated for 4 h at 37 °C. At the end of incubation, the supernatants were removed and 150 ml of DMSO (Sigma) was added to each well. The absorbance value (OD) of each well was measured at 490 nm.

### Cell proliferation analysis

Cell proliferation was analyzed using Cell Counting Kit-8(CCK-8) (Dojindo, Japan) following the manufacturer’s recommendation. Briefly, the cells were inoculated into 24-well plates with 5 × 10^3^ cells per well, and each group had 4 duplicate wells. 10 μl CCK-8 was added to the culture medium and incubated for 4 h at 37 °C. The absorbance at 450 nm was measured.

### Colony formation assay

Cells were trypsinzed and resuspended in RPMI-1640 medium supplemented with 10% FBS. An appropriate number of cells were plated into each 60 mm dish and 3 parallel dishes were scored for each treatment. After incubating for 13 days post-irradiation, cells were fixed with 100% methanol and stained with 0.5% crystal violet. Colonies containing ≥50 cells were counted as survivors. Survival curve parameters were determined by a linear-quadratic equation.

### Dual-Luciferase reporter assay

The 3′-UTR of c-Myc mRNA segment containing predicted target site of miR-449a and c-Myc-3′-UTR segment with mutated target site of miR-449A were chemically synthesized from GeneChem (Shanghai, China) (Supplementary Table S5). These segments were annealed and inserted into the Hind III and Xba I sites of pGL3-control vector (Promega, USA) and identified by DNA sequencing assay. Transfection was performed with Trans IT^®^-2020 Transfection Reagent (Mirus Bio LLC, USA) according to manufacturer’s protocol. After 48 h transfection, the cells were lysed and the luciferase activities in the lysate were measured by the Dual Luciferase Reporter Assay System (Promega, USA).

### Cell cycle analysis

Cells were exposed to 4Gy of X-rays, and were harvested 24 h after IR and fixed with 75% ethanol overnight at 4 °C. The fixed Cells were centrifuged at 2000 × g for 5 min, then washed twice in PBS and resuspended in 500 μl PI staining solution (30 μg/mL PI, 300 μg/mL RNase A in PBS) for 30 min. The DNA content of labeled cells was acquired using FACS Calibur flow cytometer (BD, USA). Cell cycle distribution was analyzed with FlowJo (Version 7.6.1).

### Western blotting

Total protein was extracted from cells using RIPA lysis buffer (Beyotime, China) containing protease inhibitors. Proteins were separated by 10% SDS-PAGE and transferred to a methanol activated PVDF membrane. The membrane was blocked for 2 h in PBST and subsequently probed with primary antibody against c-Myc (Abcam, UK), Cdc2, p-Cdc2, Cyclin B1 or Cdc25A (Cell Signaling, USA) at 4 °C overnight. β-actin served as a loading control (Bioworld, China). Membranes were washed three times for 5 min with PBST and were incubated with HRP-conjugated secondary antibody 1 h. Reactive proteins were visualized using a chemiluminescence kit (Millipore, Germany).

### Xenograft studies

Six-week-old BALB/c male nude mice were purchased from Institute of Labo-ratory Animal Sciences, CAMS and PUMC (Beijing, China). Tumorbearing mice were maintained under specific pathogen-free (SPF) conditions in Experimental Animal Center of Gansu University of Traditional Chinese Medicine(TCM). All animal experiments were conducted in accordance with the Guide for Care and Use of Laboratory Animal and all experimental protocols were approved by the Animal Ethics Committee of Gansu University of TCM. To form prostate cancer xenografts in nude mice, LNCaP cells and GV214-miR-con or GV214-miR-449a-transfected LNCaP cells (1 × 10^7^) were suspended in a 1:1 mixture of Matrigel (BD Biosciences) and culture medium, and were subcutaneously injected under aseptic conditions into at the back space. 14 days later, Tumors sites were subsequently exposed to a single dose of 4 Gy X-ray radiation by a Faxition 43885D X-ray machine. Tumor size was measured every other day using calipers and tumor volume calculated according to the equation: (long axis × short axis^2^)/2. The xenograft tumors were removed on the 12th day after radiation, and weighted after dissection.

### Statistical analysis

All statistical analyses were performed using SPSS 12.0 computer software (SPSS Inc., Chicago, IL, USA). The data are presented as the mean ± S.D. Statistical significance of the results was determined by a one-way classification ANOVA. P values < 0.05 was considered statistically significant.

## Additional Information

**How to cite this article**: Mao, A. *et al.* MicroR-449a enhances radiosensitivity by downregulation of c-Myc in prostate cancer cells. *Sci. Rep.* **6**, 27346; doi: 10.1038/srep27346 (2016).

## Supplementary Material

Supplementary Information
