# Supplementary material for: MicroRNA-449a enhances radiosensitivity by downregulation of c-Myc in prostate cancer cells
Source: Sci Rep. 2016 Jun 2;6:27346. doi: 10.1038/srep27346 (PMC4890029; doi:10.1038/srep27346)
Supplement: Supplementary Information [file srep27346-s1.pdf]

MicroR-449a enhances radiosensitivity by downregulation of c-Myc in prostate cancer cells

Aihong Mao[1, 2, 3, 6], Qiuyue Zhao[1, 4, 5, 6], Xin Zhou[1, 4, 5], Chao Sun[1, 4, 5],

Jing Si[1,4,5], Rong Zhou[1,4,5], Lu Gan[1,4,5], Hong Zhang[1,4,5※]

Supplementary tables

Table S1 Primer sequences of miR-449a, b, c and RNU6

Gene		Sequence
miR-449a		5'-TGGCAGTGTATTGTTAGCTGGT-3'
miR-449b		5'-AGGCAGTGTATTGTTAGCTGGC-3'
MiR-449c		5'-TAGGCAGTGTATTGCTAGCGGCTGT-3'
RNU6	F<br>R	5'-CTCGCTTCGGCAG CACA-3'<br>5'-AACGCTTCACGAATTTG-3'

Table S2 Primer sequences for c-Myc and GADPH

Gene		Sequence
c-Myc	F<br>R	5'-GCAGCTGCTTAGACGCTGGA-3'<br>5'-CGCAGTAGAAATACGGCTGCAC-3'
GADPH	F<br>R	5'-TCGCTCTCTGCTCCTCCTGTTC-3'<br>5'-CGCCCAATACGACCAAATCC-3'

Table S3 miRNA/Oligonucleotide sequences for expression miR-449a inserted GV214 vector

Gene	Sequence
miR-449a	5’-CGGATCCATTGTTAGCTGGCTGCTTGGGTCAAGTCAGCAGCCACA ACTACCCTGCCACTTGCTTCTGGATAAATTCTTCTTGTCAATGAAGTG CTCTGGATACCTGTGTGTGATGAGCTGGCAGTGTATTGTTAGCTGGTT GAATATGTGAATGGCATCGGCTAACATGCAACTGCTGTCTTATTGCATA TACAATGAACATCAGAGTGTAACTGAATCTGTAATTAGTGTGTGTTTA TGTGTACTTTCTGCTATGAAGCAAGCT-3’
miR-con	5’-TTCTCCGAACGTGTCACGT-3’

Table S4 SiRNA sequences for targeting c-Myc

miRNA/siRNA	Sequence
Anti-miR-449a	5’-AUCGGCUAACAUGCAACUGCUG-3’
Anti-NC	5’-UCUACUGUCACUCAGUAGUUU-3’
si-c-Myc1(S1)	5'- GCUUCACCAACAGGAACUATT-3'<br>3'-TTCGAAGUGGUUGUCCUUGAU-5'
si-c-Myc2(S2)	5'- GGAAACGACGAGAACAGUUTT-3'<br>3'-TTCCUUUGCUGCUCUUGUCAA-5'
si-c-Myc3(S3)	5'- CCACACAUCAGCACAACUATT-3'<br>3'-TTGGUGUGUAGUCGUGUUGAU-5'
NC	5'- UUCUCCGAACGUGUCACGUTT-3'<br>3'-TTAAGAGGCUUGCACAGUGCA-5'

Table S5 Oligonucleotide sequences for c-Myc-3’UTR and c-Myc-3’UTR mutant

Oligonucleotide	Sequence
c-Myc-3’UTR	<u>TCTAGA</u>ATGTCCTGAGCAATCACCTATGAACTTGTTTCAAATGCA<br>TGATCAAATGCAACCTCACAACCTTGGCTGAGTCTTGAGACTGA<br>AAGATTTAGCCATAATGTAAACTGCCTCAAATTGGACTTTGGGCA<br>TAAAAGAACTTTTTTATGCTTACCATCTTTTTTTTTTCTTTAACAG<br>ATTTGTATTTAAGAATTG<u>TCTAGA</u>
c-Myc-3’UTR MUT	<u>TCTAGA</u>ATGTCCTGAGCAATCACCTATGAACTTGTTTCAAATGCA<br>TGATCAAATGCAACCTCACAACCTTGGCTGAGTCTTGAGACTGA<br>AAGATTTAGCCATAATGATGTGACGGTCAAATTGGACTTTGGGCA<br>TAAAAGAACTTTTTTATGCTTACCATCTTTTTTTTTTCTTTAACAG<br>ATTTGTATTTAAGAATTG<u>TCTAGA</u>

Supplementary figures and figure legends

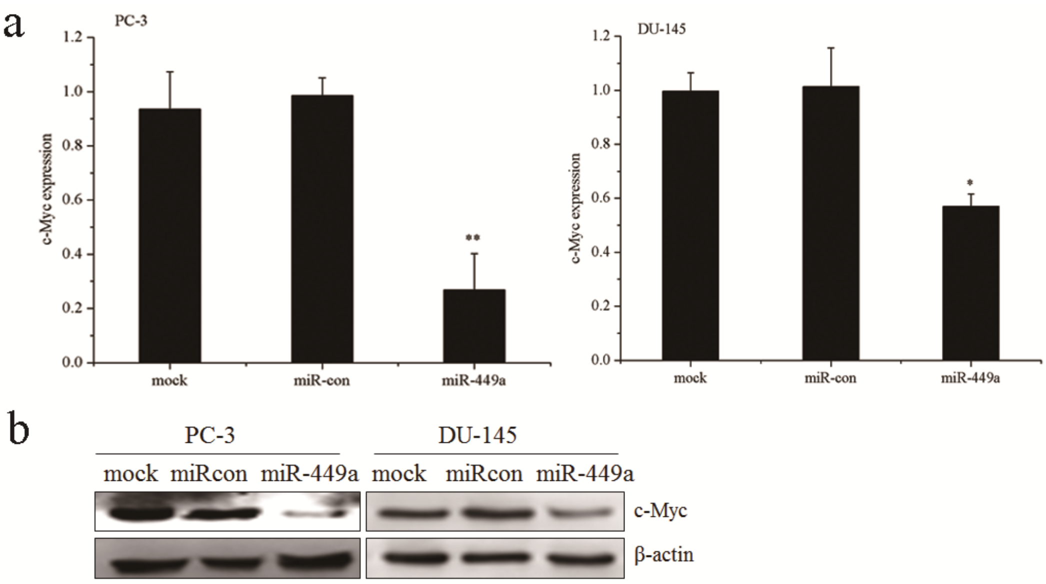

Figure S1. c-Myc was downregulated by miR-449a in prostate cancer cells. (a) The effect of miR-449a on c-Myc mRNA expression in PC-3and DU-145 cells. versus control. **(b)** The effect of miR-449a on c-Myc protein expression in PC-3and DU-145 cells. *P < 0.05 and **P < 0.01.

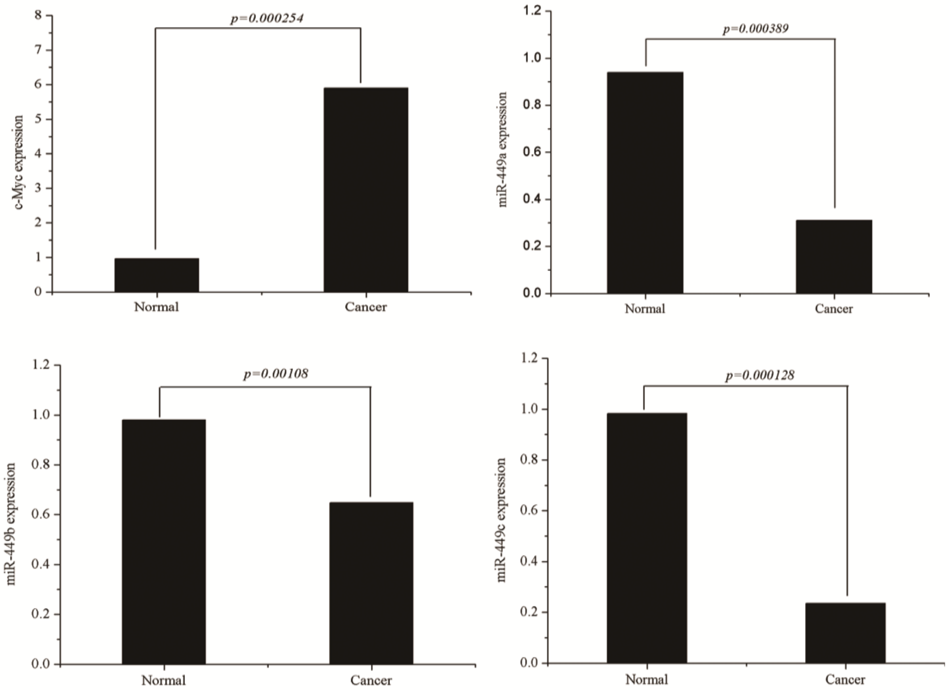

Figure S2. c-Myc and miR-449a/b/c were expressed in prostate cancer tissues. Real-time RT-PCR analysis of mature miR-449a/b/c andc-Myc expression in prostate cancer and normal prostatic tissues prepared from the same patients.

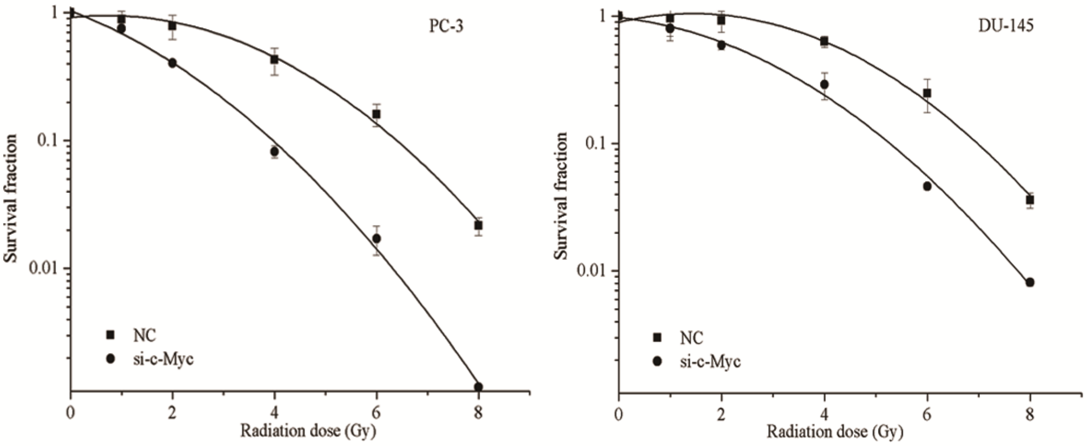

Figure S3. Knockdown of c-Myc enhanced the radiosensitity of PC-3 and DU-145 cells to IR.

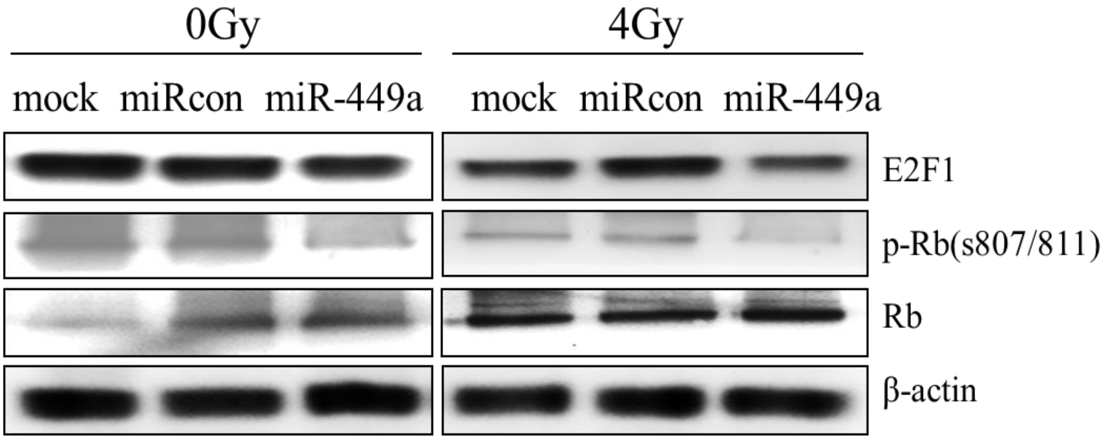

Figure S4. MiR-449a regulated Rb/E2F1 and Rb phosphorylation in response to IR.
